# Chemical Rescue and Inhibition Studies to Determine the Role of Arg301 in Phosphite Dehydrogenase

**DOI:** 10.1371/journal.pone.0087134

**Published:** 2014-01-31

**Authors:** John E. Hung, Emily J. Fogle, Neha Garg, Jonathan R. Chekan, Satish K. Nair, Wilfred A. van der Donk

**Affiliations:** 1 Department of Chemistry, University of Illinois at Urbana-Champaign, Urbana, Illinois, United States of America; 2 Department of Biochemistry, University of Illinois at Urbana-Champaign, Urbana, Illinois, United States of America; 3 Center for Biophysics and Computational Biology, University of Illinois at Urbana-Champaign, Urbana, Illinois, United States of America; 4 Howard Hughes Medical Institute, University of Illinois at Urbana-Champaign, Urbana, Illinois, United States of America; Institute of Enzymology of the Hungarian Academy of Science, Hungary

## Abstract

Phosphite dehydrogenase (PTDH) catalyzes the NAD^+^-dependent oxidation of phosphite to phosphate. This reaction requires the deprotonation of a water nucleophile for attack on phosphite. A crystal structure was recently solved that identified Arg301 as a potential base given its proximity and orientation to the substrates and a water molecule within the active site. Mutants of this residue showed its importance for efficient catalysis, with about a 100-fold loss in *k*_cat_ and substantially increased *K*_m,phosphite_ for the Ala mutant (R301A). The 2.35 Å resolution crystal structure of the R301A mutant with NAD^+^ bound shows that removal of the guanidine group renders the active site solvent exposed, suggesting the possibility of chemical rescue of activity. We show that the catalytic activity of this mutant is restored to near wild-type levels by the addition of exogenous guanidinium analogues; Brønsted analysis of the rates of chemical rescue suggests that protonation of the rescue reagent is complete in the transition state of the rate-limiting step. Kinetic isotope effects on the reaction in the presence of rescue agents show that hydride transfer remains at least partially rate-limiting, and inhibition experiments show that *K*_i_ of sulfite with R301A is ∼400-fold increased compared to the parent enzyme, similar to the increase in *K*_m_ for phosphite in this mutant. The results of our experiments indicate that Arg301 plays an important role in phosphite binding as well as catalysis, but that it is not likely to act as an active site base.

## Introduction

Phosphite dehydrogenase (PTDH), first discovered in *Pseudomonas stutzeri* WM88 [1], catalyzes the oxidation of phosphite to phosphate, which is coupled to the reduction of NAD^+^ to NADH. The thermodynamics of the reaction are 15 kcal/mol exergonic [2], and hence PTDH has been adapted for use in cofactor regeneration strategies [3]. PTDH mutants have been engineered with increased thermostability [4] and relaxed cofactor specificity [5], resulting in a stable, highly active enzyme termed 17X-PTDH that is capable of regenerating NADPH in addition to NADH. PTDH with dual cofactor specificity has been fused to flavin monooxygenases and the resulting self-sufficient biocatalysts have excellent activities for enantioselective catalysis [6]–[9]. PTDH has also found use in fluorescence assays to quantify phosphite concentrations in plants [10].

Hydride transfer is fully rate-limiting in the reaction catalyzed by either wild-type PTDH or 17X-PTDH, as determined by kinetic isotope effects and pre-steady state experiments [11], [12]. The enzyme follows an ordered kinetic mechanism with NAD^+^ binding before phosphite [1]. A number of questions about the chemical mechanism have emerged, including the identity of the catalytic base responsible for deprotonating the water nucleophile.

**Figure 1:**
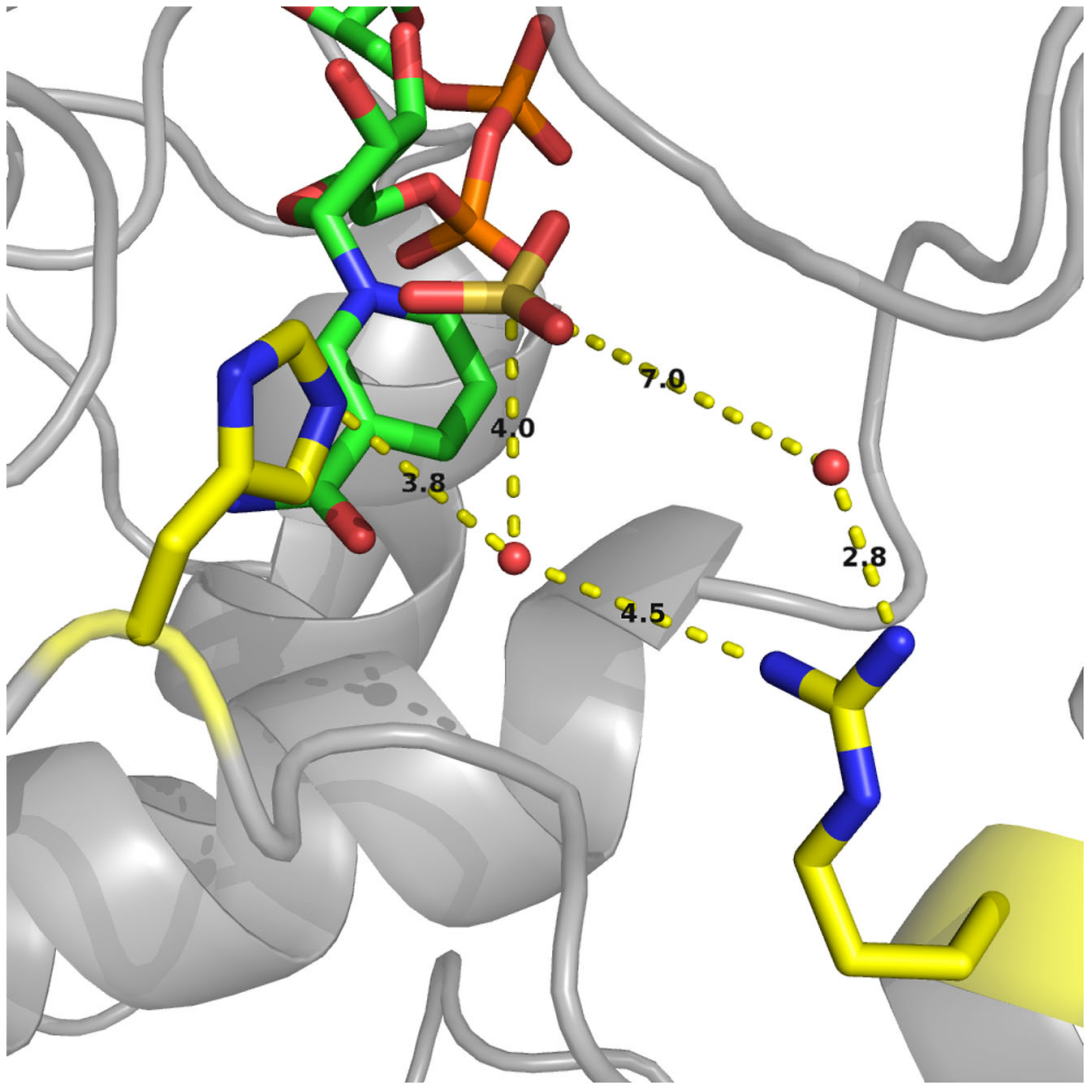
Depiction of the active site PTDH-NAD^+^-Sulfite ternary complex. The active site of the thermostable PTDH in complex with NAD^+^ and the competitive inhibitor sulfite [17], depicting the potential active site bases His292 and Arg301.

Recent reports have shown that a variety of organisms encode enzymes with PTDH activity [13]–[16]. In addition, the crystal structure of the thermostable PTDH mutant was recently published [17]. These studies have shown that a number of residues in the enzyme active site are highly conserved among the PTDH family but not in the greater family of D-hydroxy acid dehydrogenases that PTDH belongs to. Arg301 is one such residue and it is in a position in the crystal structure that might allow it to act as the catalytic base to deprotonate water for attack on phosphite (Figure 1) [17].

Arg301 is fully conserved among PTDHs, which possess moderate overall sequence identity (39–72%) [13]. Mutation of this residue to alanine (R301A) led to a ∼100-fold decrease in *k*_cat_ and a ∼500-fold increase in *K*_m,phosphite_, indicating that it plays an important role in catalysis [13]. In contrast, the Arg301Lys mutant (R301K) exhibited a slightly higher *k*_cat_ than the parent 17X-PTDH, and a relatively modest ∼20-fold increase in *K*_m,phosphite_. In light of the mutagenesis and crystallographic studies, Arg301 was hypothesized to play a role in binding and orientation of the phosphite substrate, along with possibly promoting catalysis via electrostatic interactions [13]. A role as catalytic base could not be unambiguously established or refuted. His292, which is essential for activity [12], [18], was tentatively assigned as the catalytic base [13], but no direct evidence is available to support this assignment.

In this study, we further analyzed the role of Arg301 through sulfite inhibition studies and rescue of R301A activity by addition of exogenous guanidinium analogs. In addition, X-ray crystal structures of R301A and R301K were obtained.

## Materials and Methods

### Materials

All chemicals were obtained from Sigma or Aldrich and used without further purification.

### Methods

The concentration of NAD^+^ stock solutions were determined by the absorbance at 260 nm (ε = 18,000 M^−1^ cm^−1^). The concentration of stock solutions of phosphite was determined by running the PTDH reaction to completion in the presence of excess NAD^+^. The maximum absorbance at 340 nm was then measured, which represents the concentration of NADH formed (ε = 6.2 mM^−1^ cm^−1^) and is equivalent to the concentration of phosphite consumed. All mutants were generated from the thermostable 17X-PTDH mutant as the parent enzyme as described in [13], unless otherwise noted. Expression and purification of the mutants was carried out as previously reported [13].

#### Preparation of His292 mutant constructs

The H292A mutant was made in wild-type PTDH (wt-PTDH) background using the mega primer PCR method [19]. The mega-primer was made using the following primer pair: 5′-GCT GTT CAC TCC GGC CAT AGG GTC G-3′ (with the mutated codon underlined) and 5′-GCA CGT CGA TGG ATC CTT ATC AAC ATG CGG CAG GCT CG-3′. The mega-primer obtained was used with the following primer to construct the full length gene: 5′-CGT CGA AAT TAA TAC GAC TCA CTA TAG GGG AAT TGT GAC-3′. The full length gene was then digested with NdeI and BamHI and ligated into pET15b. The H292G mutant was made using the QuikChange protocol (Agilent). The primers used to introduce the desired mutation were 5′-GCT GTT CAC TCC GGG CAT AGG GTC GGC AGT GC-3′ and 5′-GCA CTG CCG ACC CTA TGC CCG GAG TGA ACA GC-3′ (with the mutated codon underlined). Once the mutant genes were constructed, they were sequenced in their entirety to ensure that the desired mutation was incorporated and no other mutations were generated.

#### Protein crystallography

To remove the His_6_-affinity tag, thrombin (MP Biomedicals, 1 U/mg PTDH) was added to the Ni^2+^-affinity purified protein during overnight dialysis at 4°C. The cleavage product was purified by FPLC with a Superdex 200 gel filtration column (GE Healthcare) in 20 mM HEPES with 100 mM KCl (pH 7.5). The cleaved protein was determined to be >95% pure by SDS-PAGE and FPLC analysis and full activity was observed for each His_6_-tag-cleaved mutant by steady-state kinetic assays.

Crystals of PTDH variants R301A and R301K were grown using the hanging drop method. Briefly, following gel-filtration, samples of each variant were concentrated to 10 mg/mL, incubated with 10 mM NAD^+^ for 30 min, and then equilibrated against a reservoir of 0.1 M NaCl, 0.1 M sodium cacodylate pH 5.8, 16% PEG 6000 (w/v) (for R301K) or 0.1 M ammonium acetate, 0.1 M Bis Tris pH 5.5, 17% PEG 10000 (w/v) (for R301A). Crystallization samples were incubated at 288 K for 5–7 days. Prior to data collection, crystals of each PTDH variant were briefly immersed into a solution composed of the crystallization media supplemented with 20% (v/v) glycerol and vitrified by direct immersion into liquid nitrogen. Diffraction data were collected at an insertion device synchrotron source (beam line 21 ID LS-CAT at Argonne National Labs) using MAR CCD detectors. Data were integrated, merged and scaled using the HKL2000 [20] package. Crystallographic phases were solved by molecular replacement (with the PHASER [21] software as implemented in PHENIX [22] suite) using the atomic coordinates of the PTDH thermostable variant. Convincing solutions could only be obtained using the coordinates of each of the two domains for independent searches. Successive iterations of manual building were interspersed with rounds of crystallographic refinement using REFMAC [23]. Solvent molecules were judiciously placed based on chemical criteria and only retained if they resulted in a decrease in the free R factor values [24]. Final data collection and crystallographic statistics are summarized in Table S1. PDB accession numbers: 4NU5 (for PTDH R301A) and 4NU6 (for PTDH R301K).

#### Steady state kinetic assays

Initial rates were determined using a Cary 4000 UV-vis spectrophotometer (Agilent) to monitor the rate of formation of NADH using its absorbance at 340 nm (ε = 6.2 mM^−1 ^cm^−1^). Typical reactions were performed in 100 mM MOPS pH 7.25, 0.1–5 µM PTDH, holding NAD^+^ at saturating concentrations of 4 mM [13], and varying the concentration of phosphite. Reaction mixtures were allowed to equilibrate at 25°C, as verified using a thermocouple, and the reaction was initiated by addition of enzyme. Initial rates were obtained over the course of one minute. The data was fit to the Michaelis-Menten equation using OriginPro 8 (OriginLab). All experiments were performed with His_6_-tagged protein, unless otherwise noted. The His_6_-tag does not significantly alter the kinetic parameters of the enzyme [5].

#### Chemical rescue experiments with 17X-PTDH-R301A

The initial rates with and without rescue agent were determined at 25°C. Typical reactions were conducted in 100 mM MOPS pH 7.25, and contained 1 µM 17X-PTDH-R301A, 4 mM NAD^+^ (saturated), a defined concentration of rescue reagent, and varying phosphite concentrations. Control experiments showed that activity above background was only observed when enzyme, NAD^+^, phosphite and rescue agent were present. At several different concentrations of rescue reagent, phosphite concentration was varied to obtain a Michaelis-Menten curve from which the maximal activity at each concentration of rescue reagent (*k*_cat,apparent_) was extracted. The values of *k*_cat,apparent_ so determined were then plotted against the concentration of rescue reagent and fit to equation 1 [25], [26] to obtain *K*_R_, the concentration of rescue reagent at which half-maximal rate was observed, and *k*_rescue_ (*k*_cat,apparent_ under conditions of saturating rescue reagent). The apparent *K*_m,phosphite_ was determined by holding the concentration of rescue agent and NAD^+^ at saturating conditions (>10 *K*_R_) and varying the concentration of phosphite. Addition of rescue reagent at this concentration to the reaction catalyzed by the parent 17X-PTDH had no effect on activity. The compounds tested as rescue agents included acetamidine, guanidine, methylguanidine, aminoguanidine, ethylguanidine, nitroguanidine, urea, hydroxyurea, thiourea, *N*-guanylurea, methylamine, ethylamine, *n-*butylamine, and imidazole. A Brønsted plot was constructed by plotting the p*K*_a_ values of the conjugate acids of the successful rescue agents against 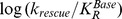
(Equation 2), where 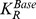 is the *K*_R_ parameter, corrected for free base concentration at pH 7.25 [26], [27], and β is the Brønsted coefficient
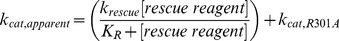
(1)

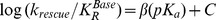
(2)


Similar chemical rescue experiments were also attempted with the inactive H292A and H292G mutants of wt-PTDH, using imidazole, 1,2,4-triazole, and methylamine at concentrations ranging between 10–100 mM, but no activity was observed.

#### pH dependence of chemical rescue

Reactions were performed at 0, 0.5, 1.0, 1.5, or 2.0 mM aminoguanidine sulfate in 100 mM MOPS at pH 7.0, 7.5, and 8.0. These concentrations were selected as they are less than ½ *K*_R_ and would be within the initial linear region of a square hyperbola for recovery of activity. NAD^+^ concentration was held at 4 mM, which was verified to be saturating at each pH value and at all concentrations of rescue reagent used. Phosphite was similarly held at saturating conditions of 1 M.

#### Kinetic isotope effects for chemical rescue experiments

Deuterium-labeled phosphite was prepared as reported previously [13]. Kinetic isotope effects (KIEs) were determined by holding NAD^+^ at saturating conditions of 4 mM and obtaining initial rates with varying concentrations of either deuterated or protiated phosphite. Experiments were performed in 100 mM MOPS (pH 7.25) at 25°C with saturating amounts (>20 *K*_R_) of rescue reagent. Apparent Michaelis-Menten constants were obtained from these data. The ratios of the apparent rate constants *k*_cat_ and *k*_cat_/*K*_m_ for labeled and unlabeled phosphite yielded ^D^ *k*_cat_ and ^D^(*k*_cat_/*K*_m_), respectively.

The overall isotope effect on the Brønsted rescue plot was determined by holding phosphite or deuterium-labeled phosphite at saturating concentrations (>10 *K*_m_) and varying the concentration of each rescue reagent. A Brønsted plot was generated for each isotopologue of phosphite and the Brønsted constant, β, was determined as described above.

#### Solvent isotope effect experiments on 17X-PTDH-R301A

All buffers and reagents were prepared in D_2_O and adjusted by addition of DCl or NaOD, according to pD = pH-reading +0.4. Sodium phosphite hexahydrate salt was dissolved in D_2_O (25 mL) to create a 0.5 M stock and the pD was adjusted to the desired value. The solution was then lyophilized and resuspended in D_2_O, and this procedure was repeated twice. The enzyme was diluted from a stock solution into 100 mM MOPS in D_2_O (1∶10 dilution of enzyme, 30 min on ice). The reactions were performed in 100 mM MOPS with 4 mM NAD^+^ at pD 7.25 and initiated by addition of 4 µM R301A-PTDH. To obtain the solvent isotope effect, the rate constants observed in D_2_O were directly compared with those obtained from the reaction performed in H_2_O at pH 7.25. Following the reaction, the stock of phosphite in D_2_O was analyzed by ^31^P NMR spectroscopy to verify no ^2^H-phosphite was formed during the incubation period.

#### Sulfite inhibition studies with 17X-PTDH-R301A

Reactions were performed in 100 mM MOPS (pH 7.25) at saturating concentrations of 4 mM NAD^+^. Sodium sulfite was adjusted to the desired concentration by serial dilution from a 0.5 M stock solution. Sulfite concentration was adjusted to 0, 50, 100, 150, 200 or 300 µM for R301A or 0, 0.5, 1, 5, 10 or 20 µM for 17X-PTDH. An experiment was also performed with 17X-PTDH-R301A and saturating concentrations of 300 mM aminoguanidine, with sulfite concentrations of 0, 100, 200, 300, 400 or 500 µM. The reaction was initiated by addition of PTDH (0.1 µM final concentration, 5 µM for R301A). At each concentration of inhibitor, phosphite concentration was varied. The data was analyzed in GraFit 7.0 [28] using the equation for competitive inhibition (Equation 3) to fit all of the data, where I is the inhibitor concentration and *K*_is_ is the dissociation constant for a competitive inhibitor [29].
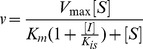
(3)


## Results

### X-Ray Crystallography of Arg301Ala and Arg301Lys Mutants of 17X-PTDH

**Figure 2:**
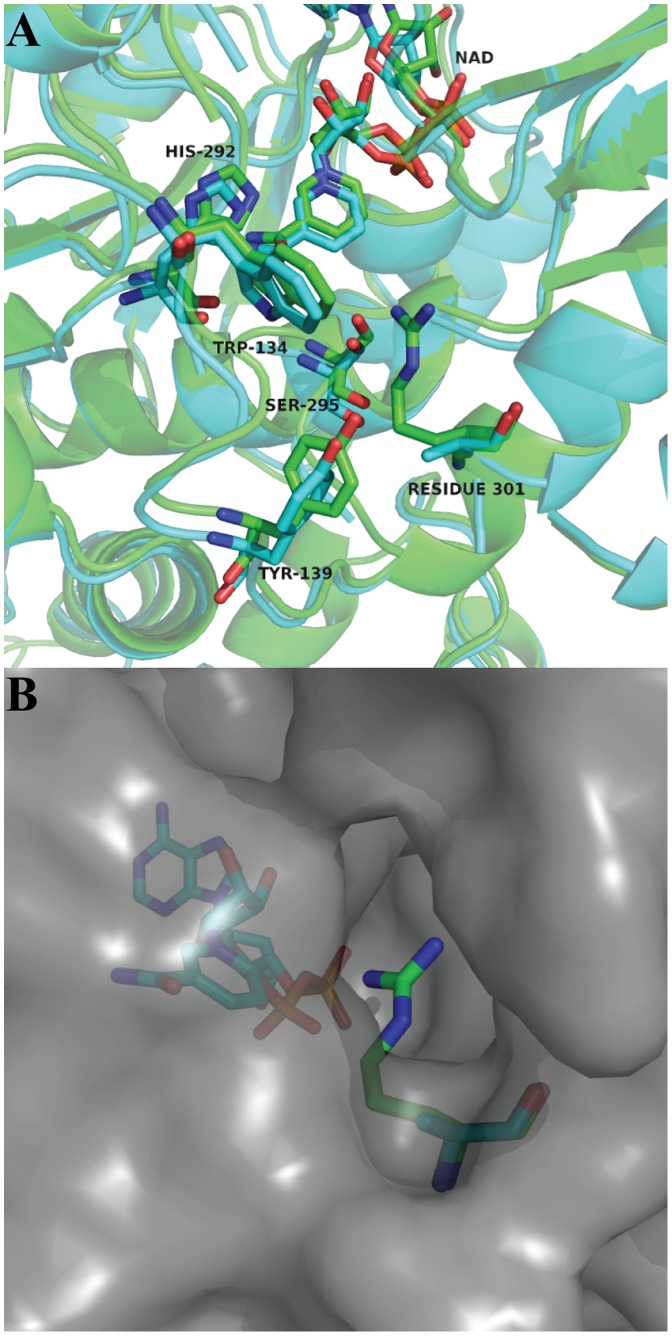
X-ray crystal structure of the R301A mutant. (A) Overlay of the crystal structures of 17X-PTDH (green) and the R301A mutant (blue) with NAD^+^ bound. Residue 301 is shown with the immediately adjacent residues Trp134, Tyr139, and Ser295, which hydrogen bond to Arg301 in the 17X-PTDH structure. Also shown is the essential residue His292. (B) Surface model of the R301A mutant (grey) overlaid with Arg301 from 17X-PTDH (green) and Ala301 from R301A (blue) in sticks, showing the presence of a solvent-accessible channel into the substrate-binding pocket. For an overlay of 17X-PTDH with PTDH-R301K, see Figure S1.

Crystals were obtained that contained NAD^+^ and that diffracted to 2.35 Å and 2.65 Å for the R301A (Figure 2A) and R301K (Figure S1) mutants, respectively. Superposition of the mutant structures with the crystal structure of the parent 17X-PTDH containing NAD^+^ [17] revealed that the overall structure of the mutants was unchanged, with the exception of the appearance of a solvent-accessible channel into the substrate binding pocket caused by the mutation of Arg301 (Figure 2B). This effect is more pronounced for R301A than R301K. Unfortunately, the side chain of Lys301 exhibits poor electron density in the latter mutant indicating that the Lys does not occupy a well-defined position in the crystal. Attempts to co-crystallize the PTDH mutants with sulfite inhibitor and NAD^+^ were unsuccessful.

### Chemical Rescue Experiments on the Arg301Ala Mutant

The multifaceted but incompletely understood role of Arg301 combined with the solvent-exposed active site in the R301A mutant prompted attempts of chemical rescue experiments [26], [27] to better define the function or Arg301. The poor activity of the R301A mutant could indeed be markedly improved by addition of exogenous guanidine, methylguanidine, acetamidine, aminoguanidine, ethylguanidine, or *N*-guanylurea (Table 1). Rescue of activity was not observed with nitroguanidine, hydroxyurea, urea, thiourea, imidazole or with primary amines such as methylamine, ethylamine and butylamine (all tested at concentrations of 20–100 mM depending on aqueous solubility limits). The failure of reagents other than guanidine derivatives to rescue mutations of Arg residues has been observed previously for other enzymes [27], [30]. For each successful reagent, the *k*_cat,apparent_ of the reaction increased in a hyperbolic fashion (Figure S2) as the concentration of rescue reagent was increased. At saturating substrate concentrations, the addition of a saturating concentration of rescue agent lead to a 10- to 30-fold increase in *k*_rescue_ compared to *k*_cat_ for the R301A mutant without any rescue reagent, yielding about 40% of wild-type activity with the most effective reagent, aminoguanidine (Table 1). Whereas *k*_cat_ could be restored, the high *K*_m,phosphite_ observed with the R301A mutant was not decreased towards wild type values in the presence of saturating rescue reagent. The activity of the parent 17X-PTDH was unaffected at the highest concentrations of rescue reagent used, suggesting that the absence of the Arg301 side chain in the mutant is necessary for the small molecules to affect activity. The stability of the engineered 17X-PTDH towards guanidinium derivatives may be related to its increased thermostability compared to wt-PTDH. The observed saturation kinetics with respect to rescue agent for the R301A mutant combined with the unperturbed kinetics of the parent 17X-PTDH in the presence of rescue agent strongly suggest that the observed improvement in catalysis by R301A imparted by the rescue reagents involves a quaternary complex (enzyme, NAD^+^, phosphite, and rescue agent).

**Table 1:** Steady state constants for chemical rescue of activity on R301A.

	*k*_rescue_ (s^−1^)	*K*_m, phosphite_ (mM)	*k*_rescue/_ *K*_m, phosphite_ (M^−1^ s^−1^)	*K*_R_ (mM)	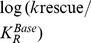	p*K*_a_^a^	^D^ *k*_cat_	^D^ *k*_cat_/*K*_m_
17X-PTDH^b^	3.2 (0.2)	0.028 (0.007)	1.2 (0.3) x 10^5^	na	na	na	2.3 (0.1)	2.1 (0.2)
R301K^b^	4.5 (0.7)	1.1 (0.1)	4.0 (0.8) x 10^3^	na	na	na	1.9 (0.1)	2.2 (0.5)
R301A^b^	0.041 (0.007)	20 (1)	2.1 (0.4)	na	na	na	2.7 (0.1)	2.1 (0.2)
R301A+guanidine	1.2 (0.1)	30 (3)	41 (4)	18 (3)	8.1 (0.1)	13.6	1.8 (0.1)	2.3 (0.1)
R301A+methylguanidine	0.68 (0.04)	130 (30)	5.2 (1.1)	55 (9)	7.2 (0.1)	13.4	1.4 (0.1)	1.6 (0.5)
R301A+acetamidine	0.86 (0.08)	130 (20)	6.7 (1.1)	63 (15)	6.3 (0.1)	12.5	1.5 (0.1)	1.8 (0.6)
R301A+aminoguanidine	1.4 (0.1)	7.4 (0.5)	180 (10)	32 (2)	5.3 (0.1)	11.0	2.1 (0.1)	3.1 (0.3)
R301A+ethylguanidine	0.52 (0.13)	nd	nd	440 (190)	6.1 (0.2)	13.3	nd	nd
R301A+*N*-guanylurea	0.42 (0.07)	nd	nd	120 (40)	−2.9 (0.2)	3.9	nd	nd

The errors given in parentheses were obtained from fits of the experimental data to the appropriate equations. na = not applicable. nd = not determined. *k*_rescue_ refers to *k*_cat_ of the enzyme in cases where no rescue reagent was added (i.e. entries 1–3). ^a^Data from reference [48].^ b^Data from reference [13].

**Figure 3:**
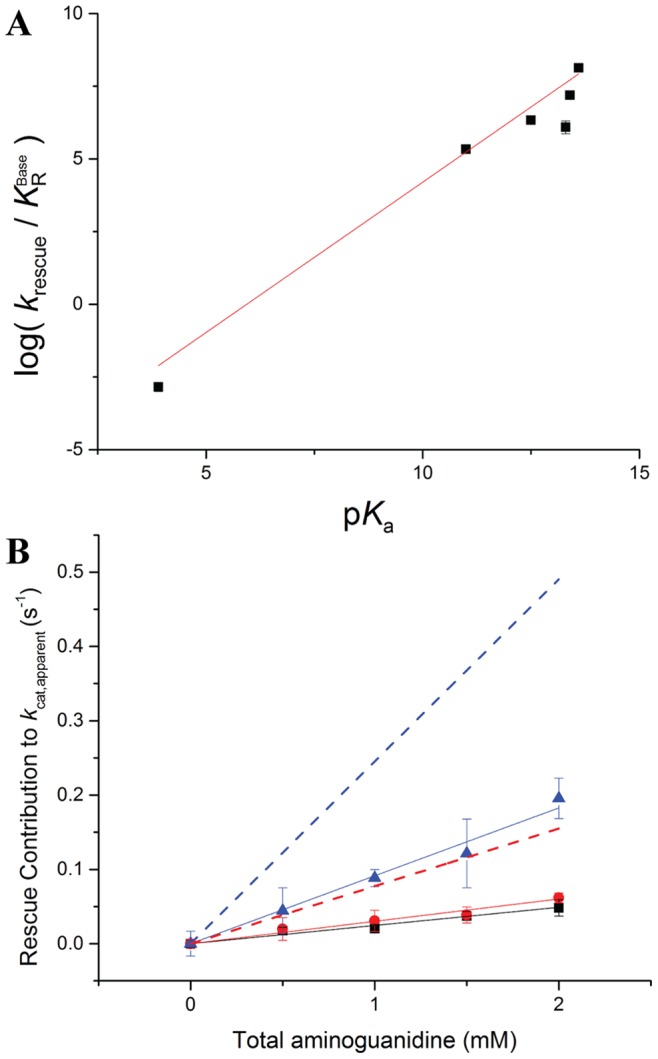
Chemical rescue plots for R301A-PTDH. (A) Brønsted plot depicting the observed rates of phosphite oxidation by R301A in the presence of various rescue reagents at pH 7.25 as a function of their p*K*_a_. β = 1.03±0.09 (r^2^ = 0.96). (B) Dependence of the rate of chemical rescue on the concentration of aminoguanidine at pH 7.0 (black squares), pH 7.5 (red circles), and pH 8.0 (blue triangles). All data was corrected for the pH-dependence of *k*_cat,R301A_ (see Figure S4). Also shown is the expected activity at pH 7.5 (red dashed line) and pH 8.0 (blue dashed line) based on the observed activity at pH 7.0 if the active reagent were the unprotonated aminoguanidine. Experiments were performed in duplicate; when error bars are not visible, the error was smaller than the marker.

The *K*_R_ value, which represents the concentration of rescue reagent that gives half-maximal rate enhancement, was adjusted to the concentration of rescue reagent in the deprotonated state at the assay pH,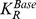, and a linear correlation was observed between the efficiency of R301A rescue and the p*K*_a_ of the conjugate acid of the rescue reagent (Figure 3A). When fit to equation 2 by linear regression, a value of the Brønsted parameter β of 1.03±0.09 was obtained (see Text S1 and Figure S3A for a discussion of the guanylurea datapoint). The β value near one suggests that transfer of a proton to the guanidine group is complete in the transition state of the rate determining step. Two scenarios can account for this observation. First, the deprotonated rescue reagent may act as a general base with complete proton transfer in the transition state of the rate determining step. Alternatively, a pre-equilibrium protonation step prior to the rate limiting step may take place, and the resulting protonated, positively-charged group is important in the rate limiting step. Because correlation between the Taft steric parameters [31] of the rescue reagents and rescue activity was poor, the steric contribution was ignored.

Substrate kinetic isotope effects (KIEs) were also determined for the assays with R301A in the presence of saturating conditions of rescue agent to determine whether the hydride transfer step remained rate limiting. Although some variation was observed in the KIEs of the R301A mutant when rescue reagents were added (Table 1), moderate KIEs were detected on both ^D^ *k*_cat_ and ^D^(*k*_cat_/*K*_m_). Hence, chemistry is at least partially rate limiting under rescue conditions. Taking into account the experimental errors, the KIEs for the rescue experiments are all quite close to those observed with 17X-PTDH.

The KIEs suggest that the hydride transfer step remains rate-limiting, as is the case in the parent 17X-PTDH [11]. We reasoned that if a large β value indeed represented full proton transfer to the guanidine group in the transition state of the rate limiting step (i.e. a late transition state with respect to proton transfer), that perhaps increasing the barrier for the hydride transfer step by using deuterium-labeled phosphite might result in a different value for β (note that the site of labeling is not the hydrogen that ends up on the guanidinium group, hence the isotope effect is not on the proton transfer step itself). However, a Brønsted plot using deuterium-labeled phosphite resulted in a β value of 1.05±0.07, which is within error of the value obtained for the protiated reagent. Hence, the rather modest change in energy barrier with deuterium-labeled phosphite did not appreciably change the proton transfer event. The solvent isotope effect (SIE) on *k*_cat_ for the wt enzyme is rather small (1.55±0.07) [12] and a similarly small SIE (1.77±0.08) was observed in this study for *k*_cat_ of the R301A mutant. Because of this small value and the difficulty to interpret small SIEs because of the many properties of the enzyme that are affected in D_2_O compared to H_2_O [32], no solvent isotope effect studies were performed for the rescue experiments.

Experiments to rescue activity for mutants of His292 were also attempted. As expected from previous results with other His292 mutants [18], the H292A and H292G mutants of wt-PTDH had no detectable activity. Unfortunately, attempts to rescue the activity of either mutant with imidazole, 1,2,4-triazole, or amine-type bases failed to result in any activity.

### pH Dependence of Chemical Rescue

As discussed above, the observed large slope of the Brønsted plot can be indicative of two different scenarios. Either a neutral guanidine group is completely protonated in the transition state of the rate determining step or the guanidine group needs to already be protonated in a step prior to the rate determining step. As has been noted previously [27], in the former case, one would expect the rate at low concentrations of rescue agent to be linearly dependent on the concentration of the unprotonated rescue reagent, whereas in the latter case, one would expect the overall rate to be dependent on the concentration of protonated rescue reagent. Thus, in favorable cases, one can distinguish between these two scenarios by determining the pH dependence of the chemical rescue rates in a pH window where the concentration of the unprotonated reagent varies strongly with pH but the concentration of the protonated reagent does not. For instance, when raising the pH from 7 to 8, the concentration of protonated aminoguanidine (p*K*_a_ = 11.0) does not change substantially, whereas the concentration of neutral aminoguanidine increases approximately 10-fold (see Table S2). Hence, if neutral aminoguanidine were the reagent that is responsible for rescue, the observed rate under conditions where phosphite and NAD^+^ are saturating should be directly linear with the concentration of neutral aminoguanidine at low concentrations, and should increase roughly 10-fold in going from pH 7 to 8 (and 3.2-fold going from pH 7.0 to 7.5, Table S2). In contrast, if protonated aminoguanidine were the active rescue reagent, then the observed rate under these same conditions should be nearly the same at pH 7 and 8. For PTDH, a complication is that at low concentrations of rescue agent, background catalysis by R301A is significant, and hence its pH dependence, reported previously [13], must also be corrected for using equation 1. Another complication is that the *K*_m_ of phosphite for R301A is strongly pH dependent [13]. Indeed, we found that at pH 8.0 the *K*_m_ value was 300±35 mM, and hence very high (i.e. molar) phosphite concentrations were needed to saturate the enzyme. In the presence of rescue agent, the *K*_m_ for phosphite decreased somewhat, but in the low millimolar range of rescue reagent, where *k*_cat,apparent_ would be linear with the concentration of rescue agent, the *K*_m,phosphite_ was still larger than 150 mM. Hence all experiments were carried out with 1 M phosphite.

The chemical rescue experiments were performed at pH 7.0, 7.5, and 8.0 with aminoguanidine as the most efficient rescue agent. At each pH value, a linear increase in *k*_cat,apparent_ was observed as the concentration of aminoguanidine was increased. At low concentrations of rescue reagent (i.e. *K*_R_>>[rescue agent]), equation 1 simplifies and *k*_cat,apparent_ corresponds to (*k*_rescue_/*K*_R_)[rescue agent]+*k*_cat,R301A_. As expected, after correction of the pH dependence of the background activity by R301A, the lines nearly intersect in the absence of aminoguanidine, (Figure 3B). The slope of the line at pH 8.0 is considerably larger than at pH 7.0 with a ratio of 3.7±0.3, and the ratio of the slopes of the lines at pH 7.5 and pH 7.0 is 1.2±0.1. Both of these ratios are, however, significantly smaller than those expected if the unprotonated compound were the active rescue agent (expected ratios of 10 and 3.2, respectively, see Figure 3B). Conversely, the ratios are also larger than 1.0 (expected if the protonated aminoguanidine were the active rescue agent). The experiment was repeated with a different batch of enzyme and reagents, but these experiments resulted in similar ratios of slopes of 3.0±0.1 (pH 8.0: pH 7.0) and 1.6±0.1 (pH 7.5:pH 7.0). Hence, the experimental pH dependence of rescue activity was not definitive with respect to the protonation state of aminoguanidine that is required for rescue.

### Sulfite Inhibition Experiments

*K*_m_ values of substrates can greatly deviate from their corresponding *K*_d_ values because the *K*_m_ term typically contains many more microscopic rate constants than the *K*_d_ term. On the other hand, the inhibition constants of competitive inhibitors (*K*_is_) can provide a direct reflection of the influence of mutations on binding events. Inhibition experiments were therefore performed with R301A to determine the *K*_is_ value for sulfite (Table 2). Similarly to the large increases in *K*_m,phosphite_ that were observed for the mutant compared to the parent 17X-PTDH (Table 1), the *K*_is_ value for sulfite also greatly increased. A *K*_is_ of 0.31±0.05 µM was observed for 17X-PTDH (see Figure S5) whereas the *K*_is_ increased to 130±20 µM for R301A, corresponding to a more than 400-fold increase. This effect is comparable in magnitude with the nearly 700-fold increase in *K*_m,phosphite_. When saturating concentrations of aminoguanidine rescue reagent were added to the reaction with the R301A mutant, the *K*_is_ value of 78±8 µM was only slightly decreased compared to the value in the absence of rescue agent. Thus, addition of the rescue reagent fails to substantially recover inhibitor binding, just like it is unable to recover *K*_m,phosphite_.

**Table 2:** Sulfite inhibition in Arg301 mutants of 17X-PTDH.

	*K*_is_ (µM)
17X-PTDH	0.31 (0.05)
R301A	130 (20)
R301A+aminoguanidine	78 (8)

## Discussion

Previous mutagenesis studies demonstrated that Arg301 plays an important role in PTDH catalysis, but its precise role could not be elucidated [13]. The crystal structure of 17X-PTDH indicates that Arg301 is situated at the dimer interface, but its side chain orients inwards towards the enzyme active site (Figure 2). As such, Arg301 is in position to directly affect catalysis, through either electrostatic interactions if it is in the guanidinium form, or by acting as a catalytic base to deprotonate a water molecule if the guanidine is deprotonated. Arginine is not commonly considered in acid-base chemistry of proteins owing to its relatively high solution phase p*K*_a_, but several recent reports have documented enzymes in which arginine is believed to act as a base [33]–[43]. Arg301 also hydrogen bonds to Trp134 through a water molecule to form the entrance to the phosphite-binding region of the active site, and hence it may play a role in phosphite binding.

Comparison of the 17X-PTDH and R301A X-ray structures, both with NAD^+^ bound, shows that the mutant has a solvent exposed phosphite binding site that is covered by the side chain of Arg301 in the wild type structure. This observation provides a possible explanation for the greatly increased values of *K*_m,phosphite_ and *K*_is,sulfite_ for the mutant. The solvent exposed active site also provided the impetus for chemical rescue studies. Indeed, several exogenous guanidinium analogs were able to substantially enhance the activity of the R301A mutant. Although the R301K mutant possesses slightly higher activity than the parent enzyme [13], amine bases failed to affect the activity of the R301A mutant. It is possible that the amines tested could not attain the correct orientation in the active site cleft introduced by the mutation of Arg301, as reported for other examples of unsuccessful rescue of an Arg mutation by alkylamines [44].

The Brønsted linear free energy relationship plot resulted in a reasonably good linear fit and a β value of 1.03±0.09. When leaving out ethylguanidine, the linear fit improves slightly, but the slope does not change (Figure S3B). Similarly large β values in the literature have previously been attributed to a very high degree of proton transfer to the rescue reagent in the transition state of the rate limiting step of the reaction [27]. In this interpretation, the active form of the guanidine reagent is in the unprotonated state allowing it to act as a base. However, an alternative interpretation of a high Brønsted constant is that the reagent is protonated in a step prior to the rate determining step, again resulting in the group being fully protonated at the transition state of the rate determining step [30], [45]. The definition of the Brønsted coefficient allows for both interpretations [32], making it difficult to differentiate directly from the rescue data [45]. Unfortunately, our studies did not allow a definitive distinction between both interpretations because the pH dependence of the chemical rescue rate with aminoguanidine fell between the expected dependences for rescue by the protonated and neutral forms of the reagent. It may be that the pH dependence of the background reaction combined with the strong pH dependence of *K*_m_ for phosphite are in part responsible for the inconclusive outcome of these experiments.

Although the pH dependence of rescue unfortunately did not provide the anticipated answer, we believe that a protonated aminoguanidine as the active rescue agent (and hence a protonated Arg side chain in the wt enzyme) is the most straightforward interpretation of all our current data. The essentially complete proton transfer indicated by the β value, the very small SIE for the wt enzyme, and the previous finding that the SIE and substrate KIE on *k*_cat_ were not multiplicative for the wt enzyme, suggesting that deprotonation of the water nucleophile and hydride transfer are not in the same step (see Text S2 for a potential alternative explanation), are all in agreement with a protonation of the guanidine prior to the rate limiting step. In addition, if Arg were the base that deprotonates the water nucleophile, its removal might be expected to greatly affect the SIE, either because deprotonation would become more important in determining the overall rate, or because the reaction might require specific base catalysis. The observed SIE of 1.77, which is close to that observed with the wt enzyme, does not provide strong evidence for either. Hence, although not definitive, we tentatively conclude that Arg301 is protonated during catalysis and is not the active site base. The importance for its protonated side chain for catalysis may be to properly orient phosphite for efficient hydride transfer, to activate the water nucleophile for deprotonation, or to activate the phosphite electrophile electrostatically. This leaves His292 as the putative base. Indeed mutations of this residue completely abolish activity [18]. Unfortunately, attempted chemical rescue studies on His292 mutants to investigate this possibility in more detail in this study were unsuccessful.

Although the catalytic activity of R301A approached that of the parent 17X-PTDH when treated with guanidinium analogues, *K*_m,phosphite_ remained very large for all rescue studies even at saturating rescue agent concentration (Table 1). In addition, sulfite inhibition studies showed a large increase in the dissociation constant for binding with the R301A mutant, and sulfite binding could not be substantially improved by addition of rescue agent. These observations may account for the unsuccessful attempts to obtain a structure of the ternary complex with sulfite for the mutant. The *K*_d_ for phosphite itself can be calculated from ^D^(V/K), ^D^V and *K*_m,Phosphite_ using the method of Klinman and Matthews [46] and is 23±6 µM for 17X-PTDH and 13±2 mM for R301A, again showing a large decrease in affinity for the substrate. Collectively, these findings along with the X-ray structures illustrate that proper positioning of the side chain of Arg301 is very important for efficient capturing of phosphite [47], which cannot be recapitulated by the rescue agents for the R301A variant.

## Supporting Information

Figure S1 **X-ray crystal structure of the R301K mutant.** Overlay of the structures of 17X-PTDH (green) and PTDH-R301K (yellow). Note that Lys301 is not well defined in the electron density of the latter structure and that the best fit is shown here.
(TIF)

Figure S2 **Chemical rescue plots of R301A-PTDH with various rescue reagents.** Plots are shown with (A) guanidine, (B) methylguanidine, (C) acetamidine, (D) aminoguanidine, (E) ethylguanidine, and (F) *N*-guanylurea used as the rescue reagent. Data was fit to Equation 1 and is reported in Table 1. The values of *k*_cat,apparent_ at each concentration of rescue reagent was determined by varying the concentration of phosphite and keeping the concentration of NAD^+^ fixed at 4 mM (saturated).
(TIF)

Figure S3 **Chemical rescue plots for R301A-PTDH with certain data points removed.** Plots are shown with either the (A) guanylurea or (B) ethylguanidine data point removed. Guanylurea has a p*K*_a_ of 3.9, removal of the point (A) yields a revised β = 0.91±0.13 (r^2^ = 0.92); the outlier is ethylguanidine, which may be because of sterics. Ethylguanidine has a p*K*_a_ of 13.3, removal of the point (panel B) yields a revised β = 1.05±0.09 (r^2^ = 0.97).
(TIF)

Figure S4 **Uncorrected pH dependence of the chemical rescue plot with aminoguanidine for R301A-PTDH.** Dependence of the rate of chemical rescue on the concentration of aminoguanidine at pH 7.0 (black squares), pH 7.5 (red circles), and pH 8.0 (blue triangles) prior to correction for the background activity of R301A without rescue agent at each pH. Experiments were performed in duplicate; when error bars are not visible, the error was smaller than the marker.
(TIF)

Figure S5 **Inhibition of PTDH activity by sulfite.** Data is shown for (A)17X-PTDH, (B) R301A-PTDH, and (C) R301A-PTDH+saturating aminoguanidine. *K*_is_ values extracted from the data are reported in Table 2.
(TIF)

Table S1 **Data collection and refinement statistics for X-ray crystallography of PTDH mutants.**
(DOCX)

Table S2 **Expected proportion of aminoguanidine in unprotonated and protonated states at various pH values.**
(DOCX)

Text S1 **Discussion of the guanylurea datapoint.**
(DOCX)

Text S2 **Discussion of an alternative explanation of the large β value and small SIE.**
(DOCX)
