# Supplementary figures and images for: Chemical Rescue and Inhibition Studies to Determine the Role of Arg301 in Phosphite Dehydrogenase
Source: PLoS One. 2014 Jan 31;9(1):e87134. doi: 10.1371/journal.pone.0087134 (PMC3909101; doi:10.1371/journal.pone.0087134)

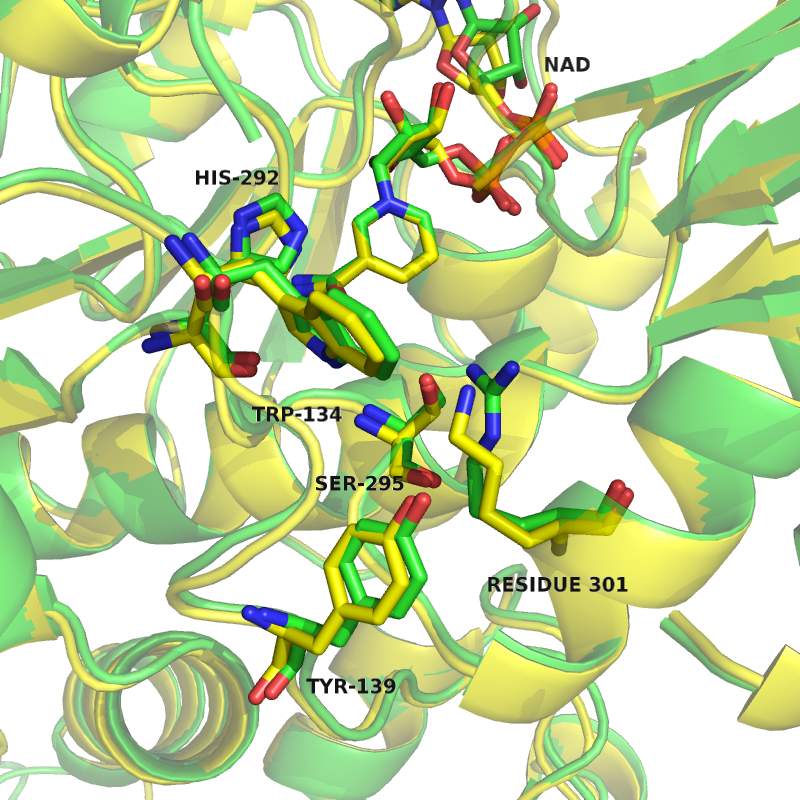

Supplement: Figure S1 — X-ray crystal structure of the R301K mutant. Overlay of the structures of 17X-PTDH (green) and PTDH-R301K (yellow). Note that Lys301 is not well defined in the electron density of the latter structure and that the best fit is shown here. (TIF) [file pone.0087134.s001.tif]

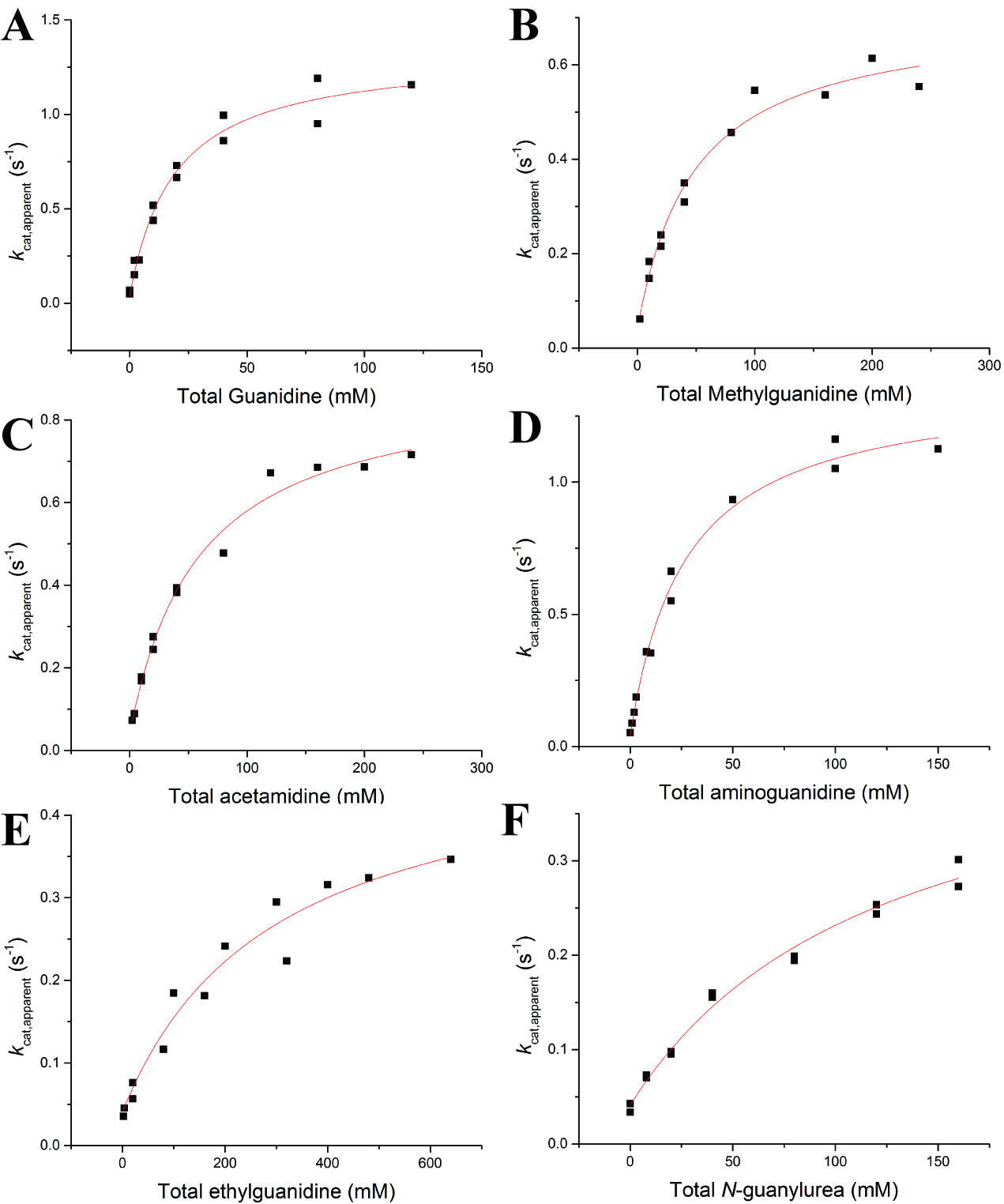

Supplement: Figure S2 — Chemical rescue plots of R301A-PTDH with various rescue reagents. Plots are shown with (A) guanidine, (B) methylguanidine, (C) acetamidine, (D) aminoguanidine, (E) ethylguanidine, and (F) N-guanylurea used as the rescue reagent. Data was fit to Equation 1 and is reported in Table 1. The values of k cat,apparent at each concentration of rescue reagent was determined by varying the concentration of phosphite and keeping the concentration of NAD+ fixed at 4 mM (saturated). (TIF) [file pone.0087134.s002.tif]

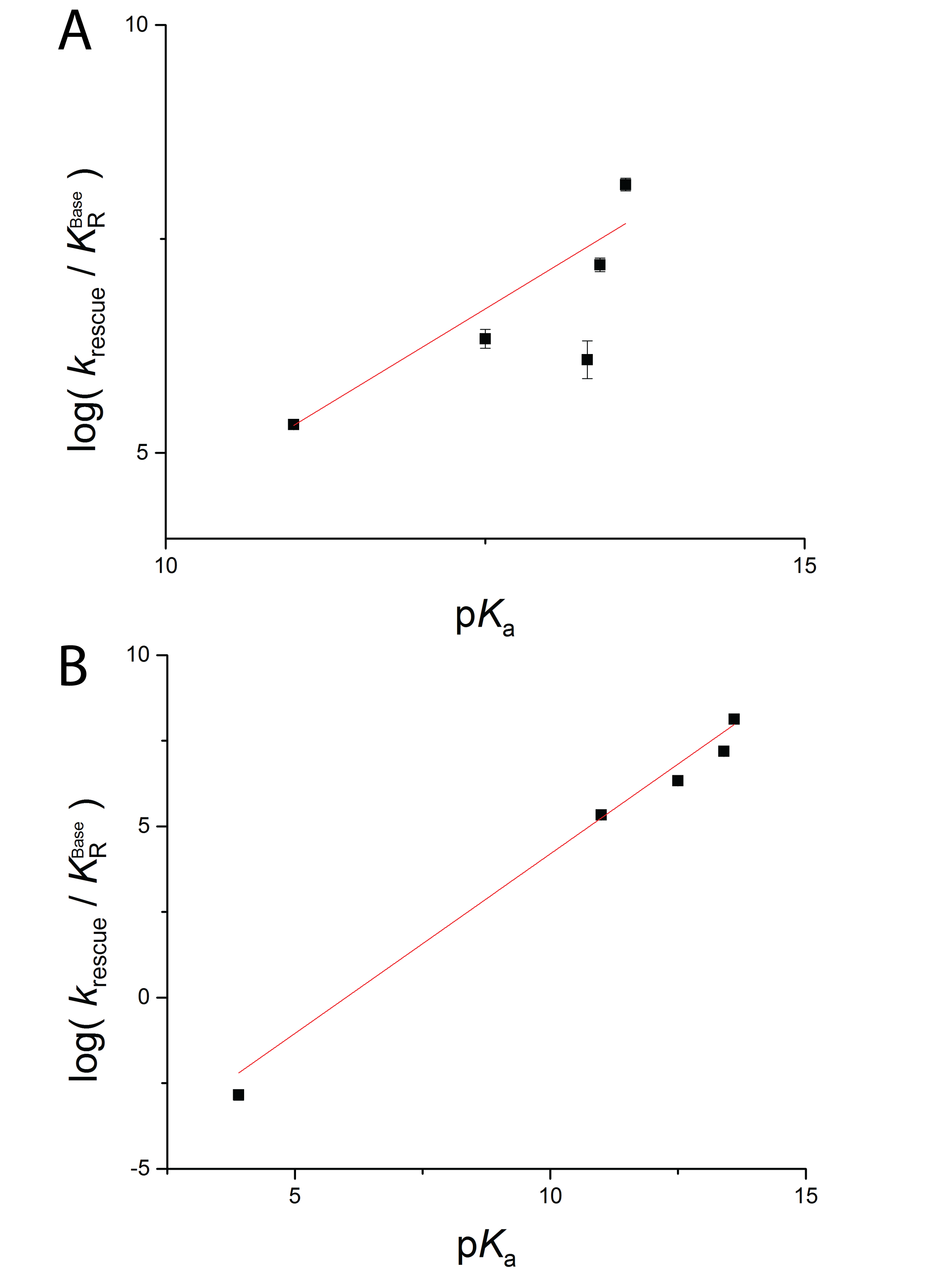

Supplement: Figure S3 — Chemical rescue plots for R301A-PTDH with certain data points removed. Plots are shown with either the (A) guanylurea or (B) ethylguanidine data point removed. Guanylurea has a pK a of 3.9, removal of the point (A) yields a revised β = 0.91±0.13 (r2 = 0.92); the outlier is ethylguanidine, which may be because of sterics. Ethylguanidine has a pK a of 13.3, removal of the point (panel B) yields a revised β = 1.05±0.09 (r2 = 0.97). (TIF) [file pone.0087134.s003.tif]

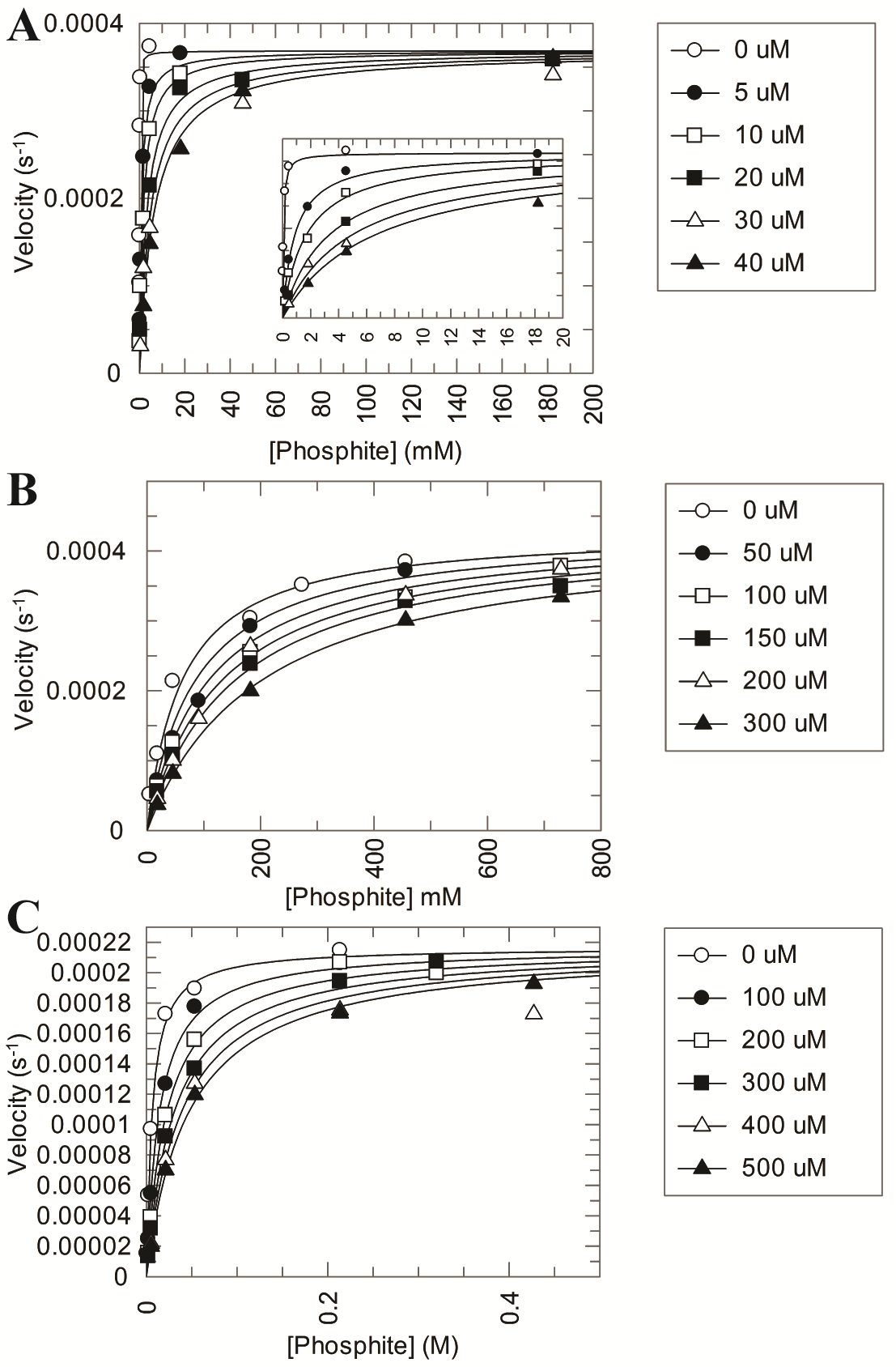

Supplement: Figure S5 — Inhibition of PTDH activity by sulfite. Data is shown for (A)17X-PTDH, (B) R301A-PTDH, and (C) R301A-PTDH+saturating aminoguanidine. K is values extracted from the data are reported in Table 2. (TIF) [file pone.0087134.s005.tif]
