# Supplementary material for: Chemical Rescue and Inhibition Studies to Determine the Role of Arg301 in Phosphite Dehydrogenase
Source: PLoS One. 2014 Jan 31;9(1):e87134. doi: 10.1371/journal.pone.0087134 (PMC3909101; doi:10.1371/journal.pone.0087134)
Supplement: Table S1 — Data collection and refinement statistics for X-ray crystallography of PTDH mutants. (DOCX) [file pone.0087134.s006.docx]

|  | R301A | R301K |
| --- | --- | --- |
| Cell Dimensions |  |  |
| Space group | P2_1_2_1_2 | P2_1_2_1_2 |
| a, b, c (Å) | 116.5, 80.5, 81.9 | 116.1, 81.5, 81.2 |
| Resolution | 50.0-2.35 (2.43-2.35) | 50.0-2.65 (2.7-2.65) |
| Total Reflections | 195,237 | 113,461 |
| Unique reflections | 30,556 | 23,441 |
| R_sym_ (%) | 9.3 (36.8) | 10.7 (66.8) |
| I/σ | 21.3 (2.9) | 13.7 (1.8) |
| Completeness (%) | 93.0 (72.7) | 99.9 (100.0) |
| Redundancy | 6.4 (4.3) | 4.9 (4.8) |
| Refinement |  |  |
| Resolution (Å) | 25.0-2.35 | 25.0-2.65 |
| No. reflections | 28,934 | 21,464 |
| R_work_/R_free_ | 18.5/23.0 | 18.1/25.5 |
| No. of atoms |  |  |
| Protein | 4,972 | 5,034 |
| NAD | 88 | 88 |
| Water | 374 | 127 |
| B-factors |  |  |
| Protein | 36.3 | 39.5 |
| NAD | 32.3 | 33.1 |
| Water | 37.3 | 33.7 |
| Ramachandran analysis |  |  |
| Favored | 89.1 | 87.7 |
| Allowed | 10.7 | 11.6 |
| Outliers | 0 | 0 |
| Rmsd |  |  |
| Bond lengths (Å) | 0.010 | 0.010 |
| Bond angels (^o^) | 1.31 | 1.62 |
|  |  |  |
