# Supplementary material for: Chemical Rescue and Inhibition Studies to Determine the Role of Arg301 in Phosphite Dehydrogenase
Source: PLoS One. 2014 Jan 31;9(1):e87134. doi: 10.1371/journal.pone.0087134 (PMC3909101; doi:10.1371/journal.pone.0087134)
Supplement: Table S2 — Expected proportion of aminoguanidine in unprotonated and protonated states at various pH values. (DOCX) [file pone.0087134.s007.docx]

|  | Unprotonated (RNH_2_) | Protonated (RNH_3_^+^) |
| --- | --- | --- |
| pH 7.0 | 0.01% | 99.99% |
| pH 7.5 | 0.03% | 99.97% |
| pH 8.0 | 0.10% | 99.90% |
| Ratio of concentrations of reagent (pH 7.5 : pH 7) | 3.2 | 1.0 |
| Ratio of concentrations of reagent (pH 8 : pH 7) | 10.0 | 1.0 |
