# Supplementary material for: Chemical Rescue and Inhibition Studies to Determine the Role of Arg301 in Phosphite Dehydrogenase
Source: PLoS One. 2014 Jan 31;9(1):e87134. doi: 10.1371/journal.pone.0087134 (PMC3909101; doi:10.1371/journal.pone.0087134)
Supplement: Text S1 — Discussion of the guanylurea datapoint. (DOCX) [file pone.0087134.s008.docx]

**Discussion of the guanylurea datapoint**

A recent computational study suggests that the major tautomer of guanylurea involves an imino form of the bridging nitrogen (i.e. that protonation occurs on the bridging nitrogen) ([*1*](#_ENREF_1)). This suggests that the p*K*_a_ value used in Figure 3 (3.9) is that of deprotonation of the central nitrogen in the guanidinium, which is quite different from the other guanidine derivatives. If rescue is affected not by the major tautomer but instead by one of the minor tautomers, then its pKa would need to be used, and the$K_{R}^{Base}$parameter would need to be adjusted. This could potentially change the slope of the line. However, since we do not know the p*K*_a_ of this tautomer, and hence we do not know its concentration at a given pH, we cannot exactly determine how this would affect the slope. Omitting the guanylurea datapoint altogether (Figure S3) still provides a line with a slope of 0.9 ± 0.1 suggesting that the discussion in the main text still holds. In addition, if it is the protonated guanylurea that affects catalysis as we suggest in this work, then the major tautomer of the neutral form is not important.

1. Kasetti, Y., and Bharatam, P. V. (2013) Pharmacophoric features of drugs with guanylurea moiety: an electronic structure analysis, *J. Mol. Model.* *19*, 1865-1874.
