# Supplementary material for: Chemical Rescue and Inhibition Studies to Determine the Role of Arg301 in Phosphite Dehydrogenase
Source: PLoS One. 2014 Jan 31;9(1):e87134. doi: 10.1371/journal.pone.0087134 (PMC3909101; doi:10.1371/journal.pone.0087134)
Supplement: Text S2 — Discussion of an alternative explanation of the large β value and small SIE. (DOCX) [file pone.0087134.s009.docx]

**Discussion of an alternative explanation of the large β value and small SIE.**

The following data is from ([1](#_ENREF_1)):

The SIE on k_cat_ for the wt enzyme is 1.55 at pH 7.25.

The substrate KIE on k_cat_ for the wt enzyme is 2.1 at pH 7.25.

A multiplicative combined SIE and substrate KIE would have been 3.25, but the observed combined SIE and substrate KIE on k_cat_ for the wt enzyme is 2.48 at pH 7.25.

This non-multiplicative combined isotope effect suggests that hydride transfer and water deprotonation occur in separate steps for the wt enzyme. However, an alternative explanation is that solvent labeling may affect more than just the water deprotonation step. If so, the observed SIE is only partially due to the deprotonation step and hence the actual isotope effect on water deprotonation may be less than 1.55. If the contribution of the water deprotonation step had an isotope effect of ~1.1, then the observed combined effect could actually be multiplicative and hence we cannot strictly rule out that water deprotonation and hydride transfer take place in the same transition state.

Such a small solvent isotope effect of 1.1 would not necessarily be inconsistent with Arg301 acting as active site base because a β value of 1 implies a very late transition state (essentially full bond formation between the guanidine nitrogen and the proton), which in turn would be expected to have a small KIE.

Hence the small SIE and non-multiplicative SIE and substrate KIE do not rule out a role of Arg301 as base, but we find this explanation less likely.

1. Relyea, H. A., Vrtis, J. M., Woodyer, R., Rimkus, S. A., and van der Donk, W. A. (2005) Inhibition and pH dependence of phosphite dehydrogenase, *Biochemistry* *44*, 6640-6649.
